# Novel approaches for the taxonomic and metabolic characterization of lactobacilli: Integration of 16S rRNA gene sequencing with MALDI-TOF MS and ^1^H-NMR

**DOI:** 10.1371/journal.pone.0172483

**Published:** 2017-02-16

**Authors:** Claudio Foschi, Luca Laghi, Carola Parolin, Barbara Giordani, Monica Compri, Roberto Cevenini, Antonella Marangoni, Beatrice Vitali

**Affiliations:** 1 Microbiology, DIMES, University of Bologna, Bologna, Italy; 2 Centre of Foodomics, Department of Agro-Food Science and Technology, University of Bologna, Bologna, Italy; 3 Department of Pharmacy and Biotechnology, University of Bologna, Bologna, Italy; Universidad Autonoma Metropolitana, MEXICO

## Abstract

Lactobacilli represent a wide range of bacterial species with several implications for the human host. They play a crucial role in maintaining the ecological equilibrium of different biological niches and are essential for fermented food production and probiotic formulation. Despite the consensus about the ‘health-promoting’ significance of *Lactobacillus* genus, its genotypic and phenotypic characterization still poses several difficulties. The aim of this study was to assess the integration of different approaches, genotypic (16S rRNA gene sequencing), proteomic (MALDI-TOF MS) and metabolomic (^1^H-NMR), for the taxonomic and metabolic characterization of *Lactobacillus* species. For this purpose we analyzed 40 strains of various origin (intestinal, vaginal, food, probiotics), belonging to different species. The high discriminatory power of MALDI-TOF for species identification was underlined by the excellent agreement with the genotypic analysis. Indeed, MALDI-TOF allowed to correctly identify 39 out of 40 *Lactobacillus* strains at the species level, with an overall concordance of 97.5%. In the perspective to simplify the MALDI TOF sample preparation, especially for routine practice, we demonstrated the perfect agreement of the colony-picking from agar plates with the protein extraction protocol. ^1^H-NMR analysis, applied to both culture supernatants and bacterial lysates, identified a panel of metabolites whose variations in concentration were associated with the taxonomy, but also revealed a high intra-species variability that did not allow a species-level identification. Therefore, despite not suitable for mere taxonomic purposes, metabolomics can be useful to correlate particular biological activities with taxonomy and to understand the mechanisms related to the antimicrobial effect shown by some *Lactobacillus* species.

## Introduction

Members of *Lactobacillus* genus are heterogeneous, Gram-positive, non-spore-forming rods or coccobacilli, catalase-negative [1]. This genus comprises close to 200 species with a G+C content usually below 50 mol% [2]. Lactobacilli are at the interface of aerobic and anaerobic life. Many lactobacilli retain the conditional capacity for respiration, but their ecology and physiology are mainly related to the fermentative conversion of sugars to organic acids, with lactic acid as the primary fermentation end product [3, 4].

The human body hosts various *Lactobacillus* species in different anatomic regions (oral cavity, gut and female genital tract) entailing different interactions with the host [5–8]. Lactobacilli play a crucial role in maintaining the ecological equilibrium of these environments, through direct antimicrobial effects, enhancement of mucosal barrier integrity, and immune modulation [9]. In addition, lactobacilli are important bacteria in food microbiology and human nutrition due to their contribution to fermented food production and their use as probiotics in food and pharmaceuticals [10, 11]. Probiotics are defined as “live microorganisms which when administered in adequate amounts confer a health benefit on the host” [12]. A number of studies have examined the role of probiotics in prevention and/or management of intestinal infections, inflammatory bowel disease and irritable bowel syndrome, respiratory tract infections, urogenital infections, periodontal disease, halitosis and allergic reactions [13].

Despite the scientific consensus about the significance of *Lactobacillus* genus for the industrial applications related to food and human health, its species’ identification still poses several difficulties. The most recent comprehensive revision of the taxonomy of the genus is based on ribosomal sequence data: for successful inclusion into the species more than 97% similarity to the consensus sequence of the 16S rRNA genes are required [14]. Although the 16S rRNA gene sequence analysis contributed to the development of a more exhaustive taxonomy for lactobacilli, it has become evident that this classification does not relate to the phenotype, impeding the correlation of phylogenetic relationships with physiological properties or ecotype [4]. In addition, 16S rRNA gene sequencing is relatively expensive, time- and labor-consuming, not suitable for routine identification [15], and, in some cases, it has insufficient discriminative power for closely related species. This implies that additional techniques, such as sequencing of more divergent protein-coding genes and/or fingerprinting techniques, should be applied to differentiate strains and allot them to the correct species after 16S rRNA gene—based clustering [4, 14].

In the last years, matrix-assisted laser desorption/ionization time of-flight mass spectrometry (MALDI-TOF MS) has proven to be a rapid and effective tool for the identification of bacteria at the species and genus levels [16]. Recently, MALDI-TOF MS has been introduced into routine microbiological diagnosis with marked success [17], and has been increasingly applied for the species identification of food associated microorganisms [18, 19]. Some attempts have been made to identify lactobacilli to species level both in clinical specimens and in food products [15, 20–25].

Unlike genotypic and proteomic techniques, validated and consolidated in microbial taxonomy studies, little information is available to date regarding the application of metabolomic methods for the identification and typing of microorganisms. Metabolomics is able to analyze different biological systems, using high-throughput analytical methods, such as nuclear magnetic resonance (NMR) spectroscopy, that allows robust and sensitive identification of metabolites produced by the cells present in the sample analyzed. Metabolites that are significantly affected by experimental variables can be identified by multivariate statistics [26, 27]. Notably, recent studies highlight the potential of metabolomics to measure the taxonomic distance between different *Lactobacillus* species and predict their anti-microbial activity [28, 29].

This study aims to evaluate the possible integration of different methodological approaches, genotypic (16S rRNA gene sequencing), proteomic (MALDI-TOF MS) and metabolomic (^1^H-NMR), for the taxonomic and metabolic characterization of *Lactobacillus*. For this purpose we used a wide selection of strains of various origin (intestinal, vaginal, food and industrial probiotic preparations), belonging to different species.

## Materials and methods

### Bacterial strains and culture conditions

A total of 40 *Lactobacillus* strains were used in this work (Table 1). MB and BC strains were isolated from fecal and vaginal samples, respectively, and belong to the collection of the Department of Pharmacy and Biotechnology (University of Bologna). DSM strains were obtained from German Collection of Microorganisms and Cell Cultures (DSMZ, Braunschweig, Germany). Seven strains were included in probiotic products (Danisco US, Madison, WI; kindly provided by Prof. Claudio De Simone).

**Table 1:** List of *Lactobacillus* strains included in the present study, genotypic identification, 16S rDNA accession numbers, MALDI TOF MS identification and source.

Strain	Genotypic identification (16S rDNA accession n.)	MALDI-TOF MS		Source
		Identification	Average score (min-max)	
**MB233**	*L*. *acidophilus* (LC155897)	*L*. *acidophilus*	2.0 (1.9–2.2)	fecal
**MB422**	*L*. *acidophilus* (LC155898)	*L*. *acidophilus*	2.1 (2.1–2.2)	fecal
**MB423**	*L*. *acidophilus* (LC155899)	*L*. *acidophilus*	1.9 (1.9–2.0)	fecal
**DSM 20079**	*L*. *acidophilus* (AB680529)	*L*. *acidophilus*	2.4 (2.2–2.4)	human
**LA14**	*L*. *acidophilus* (CP005926)	*L*. *acidophilus*	2.0 (1.9–2.1)	Danisco#
**CD2**	*L*. *brevis* (LC164743)	*L*. *brevis*	1.9 (1.9–2.0)	Danisco#
**DSM 20011**	*L*. *casei* (AF385770)	*L*. *casei*	1.8 (1.8–1.9)	cheese
**BC1**	*L*. *crispatus* (AB976542)	*L*. *crispatus*	2.3 (2.2–2.3)	vaginal
**BC3**	*L*. *crispatus* (AB976544)	*L*. *crispatus*	2.0 (1.9–2.1)	vaginal
**BC4**	*L*. *crispatus* (AB976545)	*L*. *crispatus*	2.0 (1.9–2.3)	vaginal
**BC5**	*L*. *crispatus* (AB976546)	*L*. *crispatus*	2.2 (2.1–2.2)	vaginal
**BC6**	*L*. *crispatus* (AB976547)	*L*. *crispatus*	2.1 (2.0–2.2)	vaginal
**BC7**	*L*. *crispatus* (AB976548)	*L*. *crispatus*	2.1 (1.9–2.2)	vaginal
**BC8**	*L*. *crispatus* (AB976549)	*L*. *crispatus*	2.2 (2.1–2.3)	vaginal
**DSM 20081**	*L*. *delbrueckii* subsp. *bulgaricus* (AY773948)	*L*. *delbrueckii* subsp. *bulgaricus*	2.1 (2.1–2.2)	bulgarian yoghourt
**DSM 20074**	*L*. *delbrueckii* subsp. *delbrueckii* (AY773949)	*L*. *delbrueckii* subsp. *delbrueckii*	2.1 (1.9–2.2)	sour grain mash
**DSM 20076**	*L*. *delbrueckii* subsp. *lactis* (AB680003)	*L*. *delbrueckii* subsp. *delbrueckii*	1.8 (1.8–1.9)	n.a.*
**FV13**	*L*. *delbrueckii* (LC164739)	*L*. *delbrueckii* subsp. *delbrueckii*	1.8 (1.8–1.8)	Danisco#
**BC9**	*L*. *gasseri* (AB976550)	*L*. *gasseri*	2.1 (2.0–2.2)	vaginal
**BC10**	*L*. *gasseri* (AB976551)	*L*. *gasseri*	2.0 (1.9–2.1)	vaginal
**BC11**	*L*. *gasseri* (AB976552)	*L*. *gasseri*	1.9 (1.8–2.1)	vaginal
**BC12**	*L*. *gasseri* (AB976553)	*L*. *gasseri*	2.1 (2.0–2.2)	vaginal
**BC13**	*L*. *gasseri* (AB976554)	*L*. *gasseri*	2.3 (2.2–2.4)	vaginal
**BC14**	*L*. *gasseri* (AB976555)	*L*. *gasseri*	2.3 (2.1–2.4)	vaginal
**DSM 20243**	*L*. *gasseri* (HE573914)	*L*. *gasseri*	2.1 (2.0–2.2)	human
**LB31**	*L*. *helveticus* (LC164740)	*L*. *helveticus*	2.0 (1.9–2.0)	Danisco#
**LC10**	*L*. *paracasei* (LC164738)	*L*. *paracasei* subsp. *paracasei*	1.9 (1.8–1.9)	Danisco#
**DSM 20314**	*L*. *pentosus* (D79211)	*L*. *pentosus*	2.1 (2.0–2.2)	n.a.*
**BC18**	*L*. *plantarum* (LC155900)	*L*. *plantarum*	1.9 (1.9–2.0)	vaginal
**BC19**	*L*. *plantarum* (LC155901)	*L*. *plantarum*	1.9 (1.9–2.0)	vaginal
**BC20**	*L*. *plantarum* (LC155902)	*L*. *plantarum*	2.2 (2.0–2.3)	vaginal
**DSM 20174**	*L*. *plantarum* (FR775893)	*L*. *plantarum*	2.1 (2.0–2.2)	pickled cabbage
**FV9**	*L*. *plantarum* (LC164742)	*L*. *plantarum*	1.9 (1.8–2.1)	Danisco#
**LPT**	*L*. *plantarum* (LC164741)	*L*. *pentosus*	2.1 (2.0–2.2)	Danisco#
**MB313**	*L*. *reuteri* (LC155903)	*L*. *reuteri*	2.0 (1.9–2.1)	fecal
**DSM 20016**	*L*. *reuteri* (L23507)	*L*. *reuteri*	2.1 (2.0–2.2)	intestine of adult
**B876**	*L*. *rhamnosus* (LC155904)	*L*. *rhamnosus*	2.0 (1.9–2.0)	fecal
**DSM 20021**	*L*. *rhamnosus* (D16552)	*L*. *rhamnosus*	2.0 (1.9–2.1)	n.a.*
**BC16**	*L*. *vaginalis* (AB976557)	*L vaginalis*	1.9 (1.8–2.0)	vaginal
**BC17**	*L*. *vaginalis* (AB976558)	*L*. *vaginalis*	2.0 (1.8–2.1)	vaginal

*n.a.: not available

^#^ kindly provided by Prof. De Simone

All bacterial strains were grown in Man, Rogosa and Sharpe (MRS) medium supplemented with 0.05% L-cysteine, at 37°C for 24 h in anaerobic jars supplemented with GasPak EZ. MRS and GasPak EZ were supplied by Becton Dickinson and Company (Sparks, MD).

### *Lactobacillus* fraction preparation

The turbidity of 24-h lactobacilli cultures was adjusted to an optical density (OD_600_) of 2, corresponding to a cell concentration of 5 × 10^8^ colony forming unit (CFU)/ml. Cell suspensions were centrifuged at 5,000 × *g* for 10 minutes at 4°C, then supernatants were filtered through a 0.2 μm membrane filter to obtain cell free supernatants, analysed by ^1^H-NMR to examine the extracellular metabolome. Cell pellets were washed in sterile saline and lysed in 500 μL of Enzymatic Lysis Buffer (20 mM Tris HCl pH 8, 2 mM sodium EDTA, 1.2% Triton X-100, 20 mg/mL lysozyme), incubated at 37°C for 30 min and then vortexed with 0.2 g of glass beads to ensure a complete lysis [30]. Glass beads were then precipitated by centrifugation (4,700 × *g* for 5 minutes) and the supernatants, containing cellular DNA and metabolites, were collected and employed for both DNA extraction and ^1^H-NMR analysis of the intracellular metabolome, as described below.

### DNA extraction, 16S rRNA gene sequencing and phylogenetic analysis

Genomic DNA was extracted from strains *L*. *acidophilus* MB233, MB422, MB423, *L*. *brevis* CD2, *L*. *delbrueckii* FV13, *L*. *helveticus* LB31, *L*. *paracasei* LC10, *L*. *plantarum* BC18-BC20, FV9, LPT, *L*. *reuteri* MB313, and *L*. *rhamnosus* B876. Cellular lysates were obtained from 24-h cultures as described, and total bacterial DNA was purified by using DNeasy Blood & Tissue Kit (Qiagen, Hilden, Germany).

The complete 16S rRNA gene (1.5 kb) was amplified with universal primer F27 (AGAGTTTGATCM TGGCTCAG) and R1492 (TACGGYTACCTTGTTACGACTT), as previously reported [31], and sequenced. The sequences were searched with nucleotide BLAST web service (blast.ncbi.nlm.nih.gov) to confirm the taxonomic identification at species level.

16S rRNA gene sequences of the remaining strains were available in GenBank and DDBJ Nucleotide Sequence Databases, under accession numbers reported in Table 1.

A phylogenetic tree based on 16S rDNA sequences of all 40 lactobacilli strains considered in this study was created by using MEGA 6 software [32].

### MALDI-TOF MS sample preparation and analysis

Sample preparation for MALDI-TOF MS analysis was performed as previously described, with slight modifications [33]. Cell pellets corresponding to 10^8^ CFU (24-h cultures) were washed with 300 μl of sterile water and 900 μl of absolute ethanol, then suspended in 25 μl of 70% formic acid and 25 μl of pure acetonitrile. The solutions were thoroughly vortexed and centrifuged at 18,000 × *g* for 10 minutes. Afterwards, 1 μl of the supernatants was spotted in ten replicates on a ground-steel MALDI target plate (Bruker Daltonics, Bremen, Germany), dried at room temperature and overlaid with 1 μl of MALDI HCCA matrix solution (10 mg/mL of α-ciano-4-hydroxycinnamic acid in 50% acetonitrile-2.5% trifluoroacetic acid; Bruker Daltonics). A MALDI-TOF MS measurement was performed using a Bruker Microflex MALDI-TOF MS instrument (Bruker Daltonics) operating in linear, positive ion mode and using the Flex Control 3.3 software with the following parameters: laser frequency: 20%; ion extraction delay time, 30 ns; ion source voltage one, 19 kV; ion source voltage two, 15.8 kV; and ion source lens voltage, 7.75 kV. A total of 240 laser shots was automatic acquired for each spectrum. For instrument calibration, a bacterial test standard (BTS255343; Bruker Daltonics) was used.

For species identification, spectra collected within a mass range of 2,000 to 20,000 Da were analyzed with Bruker Biotyper 3.1 software and compared with the ones of the reference database. The resulting similarity values were expressed as a log score. In particular, a score ≥ 2.0 allowed the identification at the species level, a score comprised in the range 1.7–2.0indicated identification only at the genus level, whereas any score under 1.7 meant no significant similarity of the obtained spectrum with any database entry (not reliable identification).

A clustering analysis of all the *Lactobacillus* strains, belonging to different species, was performed by the generation of a score-oriented dendrogram. In particular, the main spectrum profiles (MSPs) of each strain were generated from at least 8 technical replicates (the ones with the highest score values at the species identification) using the MALDI Biotyper 3.1 software, with default setting parameters [34]. A peak quality control was performed using FlexAnalysis software 3.3 (Bruker Daltonics): spectra with outlier peaks or anomalies were removed from the spectra set of the *Lactobacillus* strain. The relationship between MSPs obtained from each strain was visualized in a score-oriented dendrogram using the average linkage algorithm implemented in the MALDI Biotyper 3.1 software.

To evaluate the reliability of MALDI-TOF MS in *Lactobacillus* identification without a protein extraction procedure, a direct analysis of bacterial colonies was performed starting from freshly overnight cultures on MRS agar and without a detailed extraction step, as already described [21].

### ^1^H-NMR analysis

For each *Lactobacillus* strain 700 μl of cell free supernatant and 350 μl of cellular lysate were added to 160 μl of a D2O solution of 3-(trimethylsilyl)-propionic-2,2,3,3-d4 acid sodium salt (TSP) 6.25 mM set to pH 7.0 by means of a 100 mM phosphate buffer. ^1^H-NMR spectra were recorded at 298 K with an AVANCE III spectrometer (Bruker, Milan, Italy) operating at a frequency of 600.13 MHz, following the procedure previously described [27, 35]. The signals were assigned by comparing their chemical shift and multiplicity with Chenomx software data bank (Chenomx Inc., Canada, ver 8.2), with standard (ver. 10) and HMDB (ver. 2) data banks. Differences in the extracellular/intracellular metabolome composition were firstly assessed by calculating the intra-groups Euclidean distance in a multidimensional space where each dimension represented the concentration of a molecule quantified in the cell free supernatant or cellular lysate. In a second time, differences in intracellular/extracellular metabolites were calculated by means of a one-tailed unpaired Wilcoxon test, through the homonym function implemented in R computational software (www.r-project.org). A probability value for null hypothesis of 0.05 was accepted, corrected according to Bonferroni for multiple comparisons.

### Nucleotide sequence accession numbers

The nucleotide sequences of the 16S rRNA gene of the *Lactobacillus* strains sequenced in the present work (*L*. *acidophilus* MB233, MB422, MB423, *L*. *brevis* CD2, *L*. *delbrueckii* FV13, *L*. *helveticus* LB31, *L*. *paracasei* LC10, *L*. *plantarum* FV9, LPT, BC18-BC20, *L*. *reuteri* MB313, *L*. *rhamnosus* B876) have been deposited in the DDBJ nucleotide sequence database under accession numbers reported in Table 1.

## Results

### Phylogenetic characterization of *Lactobacillus* strains

**Fig 1:**
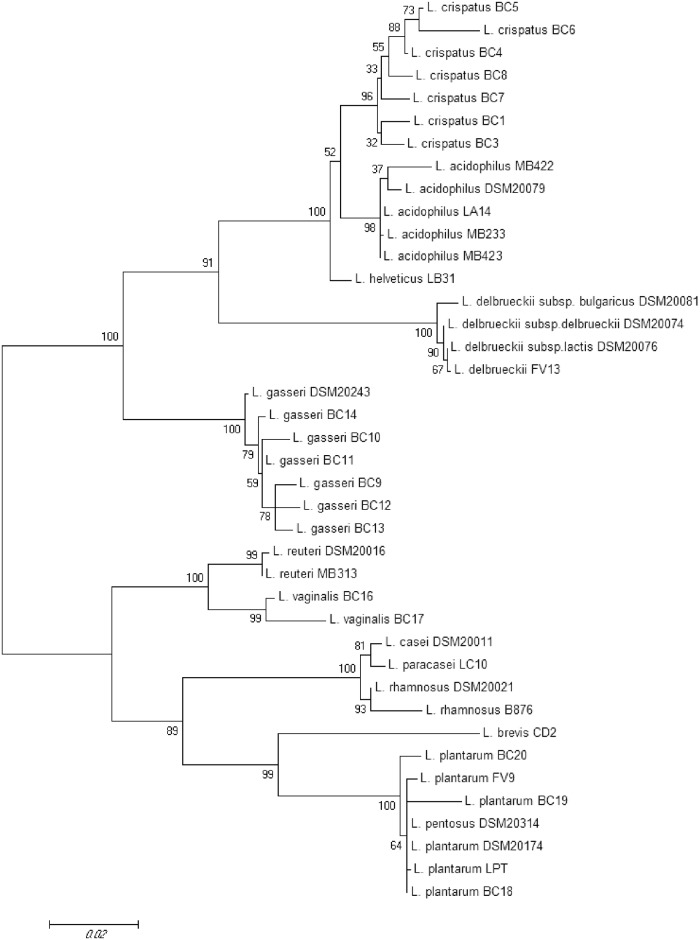
Phylogenetic tree based on lactobacilli 16S rRNA sequences. The Neighbor-Joining method was used to infer evolutionary history. The evolutionary distances were computed using the Maximum Likelihood method based on Tamura-Nei model [32]. The tree is drawn to scale, with branch lengths measured in number of substitutions per site. The bootstrap values inferred from 1000 replicates is shown next to the branches. The analysis involved 40 nucleotides sequences. All positions containing gaps and missing data were eliminated. The tree was obtained by using MEGA 6 software.

The genotypic identification of the *Lactobacillus* strains used in this work is reported in Table 1. Complete sequences of 16S rRNA gene of *L*. *acidophilus* MB233, MB422, MB423, *L*. *brevis* CD2, *L*. *delbrueckii* FV13, *L*. *helveticus* LB31, *L*. *paracasei* LC10, *L*. *plantarum* BC18, BC19, BC20, FV9, LPT, *L*. *reuteri* MB313 and *L*. *rhamnosus* B876, were amplified and sequenced (ca. 1,500 nts). For the remaining strains, complete 16S rRNA gene sequences were already available in GenBank and DDBJ Nucleotide Sequence Databases. A phylogenetic tree of lactobacilli, on the basis of the 16S rDNA sequences, was built by applying the neighbor-joining method (Fig 1). As expected, strains belonging to the same *Lactobacillus* species clustered all together, and two main groups could be identified: the first included *L*. *crispatus*, *L*. *acidophilus*, *L*. *helveticus*, *L*. *delbrueckii*, and *L*. *gasseri* species, the other comprised *L*. *reuteri*, *L*. *vaginalis*, *L*. *casei*, *L*. *paracasei*, *L*. *rhamnosus*, *L*. *brevis*, *L*. *plantarum* and *L*. *pentosus* species. Notably, *L*. *pentosus* DSM 20314 fell into *L*. *plantarum* group confirming the high phylogenetic similarity between the two species [36, 37].

### Identification of lactobacilli with MALDI-TOF MS analysis

The MALDI-TOF MS analysis of *Lactobacillus* strains performed after protein extraction with formic acid/acetonitrile showed the great potential of this technique in the taxonomic characterization of lactobacilli. For each bacterial strain, the ten technical replicates gave the same species identification with score values > 1.8 and the average score value was always ≥ 1.9, except for three strains (*L*. *casei* DSM 20011, *L*. *delbrueckii* subsp. *lactis* DSM 20076 and *L*. *delbrueckii* FV13). The analysis of bacterial colonies directly from MRS agar plates showed the same species identification obtained with the protein extraction method and no significant differences were noticed in MALDI-TOF score values (data not shown).

**Fig 2:**
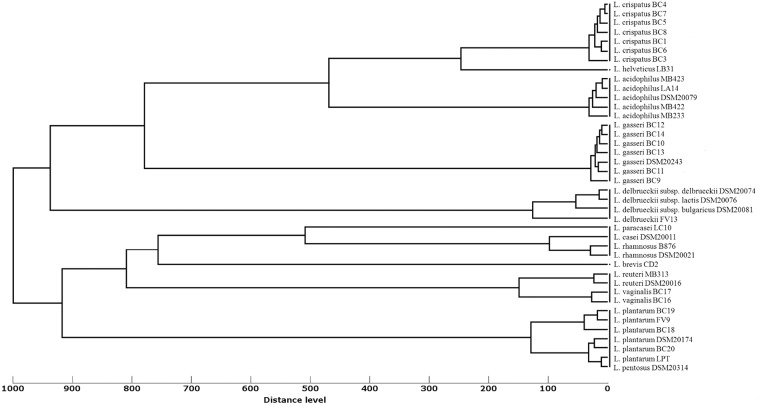
Cluster analysis of MALDI-TOF MS spectra obtained from the *Lactobacillus* strains included in the study. In the MSP dendrogram, relative distance between isolates is displayed as arbitrary units. Zero indicates complete similarity and 1,000 indicates maximum dissimilarity.

Fig 2 shows the hierarchic dendrogram of the 40 *Lactobacillus* strain MSPs created in relation to their mass signals and peak intensities. At an arbitrary distance level of 1000 (maximum dissimilarity), MSP dendrogram clustered the lactobacilli in two main groups: the first one comprised *L*. *crispatus*, *L*. *helveticus*, *L*. *acidophilus*, *L*. *gasseri* and *L*. *delbrueckii* species and the second one included *L*. *paracasei*, *L*. *casei*, *L*. *rhamnosus*, *L*. *brevis*, *L*. *reuteri*, *L*. *vaginalis*, *L*. *plantarum* and *L*. *pentosus* species. At minor distance levels, each main group was then subdivided in smaller sub-groups: for example, at a distance level of 900, *L*. *delbrueckii* cluster was clearly separated from the group including *L*. *helveticus*, *L*. *crispatus*, *L*. *acidophilus* and *L*. *gasseri*, whereas at a distance level of 700, *L*. *gasseri* group was definitely distinct from the other species. Similarly, at an arbitrary distance level of 900, the cluster including *L*. *pentosus* and *L*. *plantarum* species was separated from the group comprising *L*. *casei*, *L*. *paracasei*, *L*. *rhamnosus*, *L*. *brevis*, *L*. *reuteri* and *L*. *vaginalis* species, while at a distance level of 800 a distinct cluster with *L*. *vaginalis* and *L*. *reuteri* was noticed. At a distance level of 200, each grouping was represented by a single *Lactobacillus* species.

### Comparison of genotypic and MALDI-TOF identification of lactobacilli

When compared to the genomic analysis, MALDI-TOF MS allowed to correctly identify at the species level all the 40 *Lactobacillus* strains, except one, with an overall concordance between the two methods of 97.5% (39/40). The only discordant result was represented by *L*. *plantarum* LPT, identified as *L*. *pentosus* at MALDI-TOF MS analysis. To note, a previous characterization of this strain by automated ribotyping had revealed greater homology with *L*. *pentosus* rather than with *L*. *plantarum* [36]. Table 1 shows in details the *Lactobacillus* species identification obtained with the genomic analysis compared to MALDI-TOF MS. When the subspecies-level identification was available (three *L*. *delbrueckii* strains), MALDI-TOF MS analysis agreed with the genomic approach in two cases out of three, with the only exception of *L*. *delbrueckii* subsp. *lactis* DSM 20076 identified as *L*. *delbrueckii* subsp. *delbrueckii*. Moreover, in two cases, unlike the 16S rRNA gene sequencing that provided only species-level identification (*L*. *paracasei* LC10 and *L*. *delbrueckii* FV13), MALDI-TOF MS analysis allowed to obtain information at subspecies level (*L*. *paracasei* subsp. *paracasei* and *L*. *delbrueckii* subsp. *delbrueckii*).

### Variations in lactobacilli metabolome correlated with taxonomy

Consistently with previous reports on similar matrices [38, 39], a total of 30 and 17 molecules were identified by ^1^H-NMR analysis in the extracellular and intracellular metabolome, respectively. These metabolites mainly belong to the families of amino acids, organic acids, monosaccharides, ketones and alcohols (Table A and B, in S1 File).

For the metabolomic analysis, lactobacilli were arbitrarily subdivided in seven groupings on the basis of 16S rRNA gene sequence phylogenetic tree and MALDI-TOF MS score-oriented dendrogram. In particular, differences in the intracellular/extracellular metabolome composition were assessed for the following species groupings: *L*. *crispatus*, *L*. *gasseri*, *L*. *acidophilus*, *L*. *delbrueckii*, *L*. *plantarum-L*. *pentosus*, *L*. *reuteri-L*. *vaginalis* and *L*. *casei-L*. *paracasei-L*. *rhamnosus*. About that, it is worthy to underline that the proposed groupings were similar and comparable to others previously reported [40–42]. *L*. *helveticus* and *L*. *brevis* species were excluded from the metabolomic analysis, given that only one strain for each of these species was available.

No specific metabolic profiles related to certain species or species groupings were clearly identified. However, although the different *Lactobacillus* groupings showed the same pool of metabolites, several significant differences were noticed when considering the concentration of each molecule, both in the extracellular and in the intracellular metabolome. In order to seek correlations between taxonomy and metabolome, we created a multidimensional space, where each axis reported the concentration of a molecule quantified by ^1^H-NMR. Concerning the extracellular metabolome, for *L*. *crispatus* (*P* = 3 × 10^−3^) and *L plantarum*-*L pentosus* (*P* = 2 × 10^−18^) groupings, the intra-group distance in such space was statistically lower than the average distance among each investigated *Lactobacillus* strain. Concerning the intracellular metabolome, similar results were found for *L*. *plantarum*-*L*. *pentosus* (*P* = 2 × 10^−4^), *L*. *crispatus* (*P* = 1 × 10^−4^), *L*. *gasseri* (*P* = 3 × 10^−8^) and *L*. *delbrueckii* (*P* = 4 × 10^−4^) groupings.

To evaluate differences in the concentration of single metabolites, we performed univariate tests. This analysis allowed to identify 4 metabolites in the cell free supernatant (acetoin, acetone, pyruvate and glucose) and 8 molecules in the cellular lysate (AMP, lactate, lysine, NAD+, propionate, succinate, uracil, and valine) showing significantly different concentrations between the diverse *Lactobacillus* species groupings considered.

**Fig 3:**
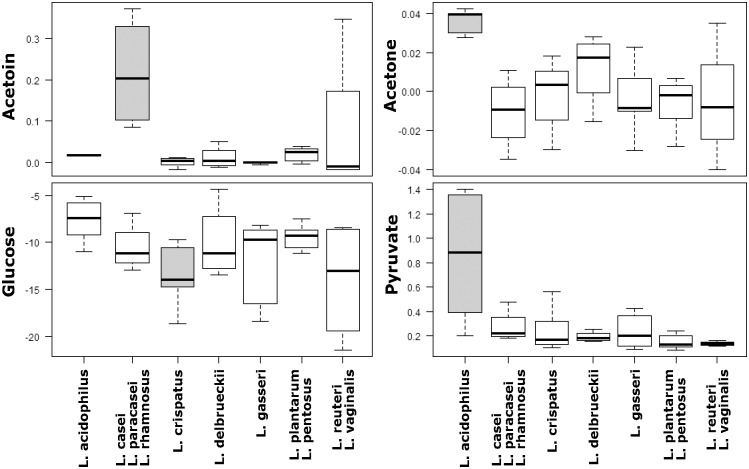
Variations in lactobacilli extracellular metabolome. Box plots represent the concentration (mM) of extracellular metabolites which vary significantly among the diverse *Lactobacillus* species considered. Metabolites were quantified in cell free supernatants by ^1^H-NMR. Lines within the boxes indicate the median values of the metabolite concentration and each box represents the interquartile range (25–75th percentile). The bottom and top bars indicate the 10th and 90th percentiles, respectively. Boxes were colored in grey to highlight the *Lactobacillus* species groupings that show significantly different concentration of the corresponding metabolite (P<0.05, Bonferroni-adjusted).

Concerning the extracellular metabolome (Fig 3), it is noteworthy to underline that *L*. *crispatus* showed the highest glucose consumption compared to the other *Lactobacillus* groupings (*P* = 1 × 10^−3^), whereas *L*. *acidophilus* species was characterized by the highest-level production of acetone and pyruvate (*P* = 3 × 10^-4^and *P* = 1 × 10^−3^, respectively). Moreover, the grouping including *L*. *casei*-*L*. *paracasei*-*L*. *rhamnosus* species differed significantly from the other species for acetoin production (*P* = 1 × 10^−5^).

**Fig 4:**
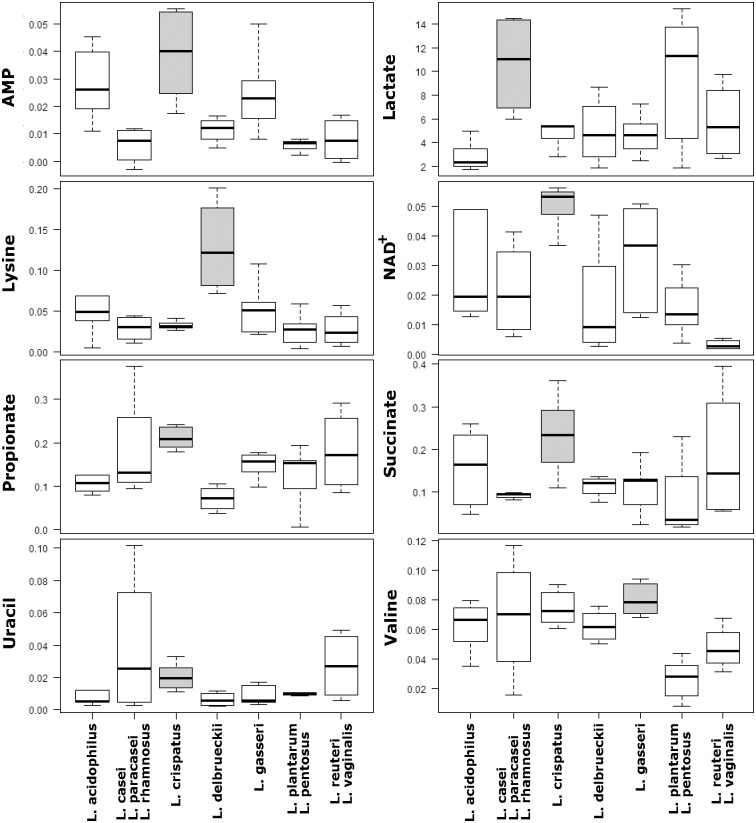
Variations in lactobacilli intracellular metabolome. Box plots represent the concentration (mM) of intracellular metabolites which vary significantly among the diverse *Lactobacillus* species considered. Metabolites were quantified in cellular lysates by ^1^H-NMR. Lines within the boxes indicate the median values of the metabolite concentration and each box represents the interquartile range (25–75th percentile). The bottom and top bars indicate the 10th and 90th percentiles, respectively. Boxes were colored in grey to highlight the *Lactobacillus* species groupings that show significantly different concentration of the corresponding metabolite (P<0.05, Bonferroni-adjusted).

The intracellular metabolome analysis (Fig 4) highlighted that *L*. *crispatus* was the largest producer of AMP (*P* = 3 × 10^−4^), NAD+ (*P* = 1 × 10^−4^), propionate (*P* = 1 × 10^−4^) succinate (*P* = 8 × 10^−4^) and uracil (*P* = 8 × 10^−5^), compared to the other species. Moreover, *L*. *gasseri* and *L*. *delbrueckii* were characterized by the highest production of valine (*P* = 2 × 10^−3^) and lysine (*P* = 1 × 10^−5^), respectively. Finally, the lactate production was the metabolic signature of *L*. *casei*-*L*. *paracasei*-*L*. *rhamnosus* grouping (*P* = 1 × 10^−3^).

The association between metabolome and taxonomy is outlined in Table 2. This table shows how the increase/decrease of a specific extracellular/intracellular metabolite is characteristic of a particular *Lactobacillus* species or grouping of species.

**Table 2:** Association between a variation (increase/decrease) of metabolites (extracellular/intracellular) and *Lactobacillus* species or grouping of species.

Metabolite	Cellular localization	Variation	Species/Grouping of species
**Acetoin**	extracellular	Increase	*L*. *casei-L*. *paracasei-* *L*. *rhamnosus*
**Glucose**	extracellular	Decrease	*L*. *crispatus*
**Acetone**	extracellular	Increase	*L*. *acidophilus*
**Pyruvate**	extracellular	Increase	*L*. *acidophilus*
**AMP**	intracellular	Increase	*L*. *crispatus*
**Lysine**	intracellular	Increase	*L*. *delbrueckii*
**Propionate**	intracellular	Increase	*L*. *crispatus*
**Uracil**	intracellular	Increase	*L*. *crispatus*
**Lactate**	intracellular	Increase	*L*. *casei-L*. *paracasei-* *L*. *rhamnosus*
**NAD**^**+**^	intracellular	Increase	*L*. *crispatus*
**Succinate**	intracellular	Increase	*L*. *crispatus*
**Valine**	intracellular	Increase	*L*. *gasseri*

## Discussion

An accurate *Lactobacillus* species identification is crucial in light of the findings that different species are able to exert diverse effects on the host. For example, it is well known that particular *Lactobacillus* species, as *L*. *crispatus*, dominate the vaginal microbiota of healthy premenopausal women, whereas other species, as *L*. *iners*, are often found in women with vaginal dysbiosis [43, 44]. Moreover, the correct species identification is fundamental in the choice of the right strain during probiotic formulation, since it has been demonstrated a high species-specificity in *Lactobacillus* activity against pathogens [28, 29, 45]. In this study, a multi-omic approach was assessed for the taxonomic and metabolic characterization of different *Lactobacillus* species: the traditional genotypic approach based on the 16S rRNA gene sequence analysis was compared and integrated with a proteomic approach based on MALDI-TOF MS ribosomal protein pattern analysis and with a ^1^H-NMR metabolomic approach focused on the bacterial intracellular and extracellular metabolome.

16S rRNA gene sequencing is regarded as an established method in taxonomic studies and is also applied for clinical diagnosis [46]. Even though this method has proved to be highly discriminative for *Lactobacillus* species identification, in some cases it fails to differentiate between closely related species or subspecies, such as *L*. *casei* and *L*. *paracasei* or *L*. *plantarum* and *L*. *pentosus*, due to the substantial similarities of their 16S rRNA gene sequences [21, 37]. Moreover, in our experience, the analysis of ribosomal sequences did not allow to discriminate between the different subspecies of *L*. *delbrueckii* and *L*. *paracasei*, as already stated [47, 48].

MALDI-TOF MS represents a simple, reliable and cost-saving tool for the rapid taxonomic characterization of lactobacilli of different origin [15, 20–25]. Up to date, the use of MALDI-TOF MS for *Lactobacillus* species identification has been particularly pointed towards the analysis of strains originated from food and probiotics [15, 22, 49, 50] and only a few studies focused on clinical isolates [21, 24, 51]. In this work, we gave particular attention to lactobacilli isolated from the human microbiota (gut/vagina), demonstrating the potential of MALDI-TOF MS to identify species that are of importance for the human health. Our study demonstrates the high discriminatory power of MALDI-TOF MS analysis for the identification of *Lactobacillus* species, as underlined by the excellent agreement with the genotypic identification. In some cases, i.e. subspecies-level identification of *L*. *delbrueckii* and *L*. *paracasei*, MALDI-TOF MS could even overcome the limits of 16S rRNA gene sequencing. The only discordant identification regarded the probiotic strain *L*. *plantarum* LPT, categorized as *L*. *pentosus* by MALDI-TOF MS. To note, a previous taxonomic characterization of this strain had revealed good homology levels with *L*. *pentosus* by automated ribotyping, whereas the 16S-23S rRNA sequence indicates *L*. *plantarum* as referee species [36]. The identification as *L*. *pentosus* by MALDI-TOF MS and ribotyping is not inconsistent and could be explained by considering the close phylogenetic relationship between *L*. *plantarum* and *L*. *pentosus* species [37, 52].

Considering that only MALDI TOF scores ≥ 2.0 are accepted for species assignment and scores between 1.7 and 2.0 are accepted exclusively for genus level interpretation, our results could seem not always convincing, i. e. when the identifications had scores < 2.0 at least in one of the replicates. However, for each bacterial strain, ten technical replicates gave always the same species identification and, when compared to the genomic analysis, MALDI-TOF MS allowed to correctly identify all the 40 *Lactobacillus* strains at the species level, except one. For these reasons, it is worth mentioning that even MALDI TOF scores in the range 1.7–2.0 could be considered acceptable for *Lactobacillus* species level identification. Nevertheless, the extension of the Biotyper reference database could probably improve MALDI-TOF MS performance in *Lactobacillus* species identification, especially for those species showing the lowest average score values (*L*. *casei* and *L*. *delbrueckii*), as already raised by other authors [15].

Moreover, when we compared the two different protocols (protein extraction versus colony-picking) of sample preparation for MALDI-TOF MS analysis, a perfect agreement in *Lactobacillus* species identification was observed. Thus, the colony-picking from agar plates can be suggested in routine clinical practice for its simplicity and few minutes’ hands-on-time. Globally, our results strongly support the role of MALDI-TOF MS in studies of lactobacilli taxonomy. In this context, the low cost, together with the ease-of-use and the rapidity of this technique are fundamental strengths compared to the more complex and expensive genotypic approaches [40, 41].

Differently from to the well-established role of genotypic and proteomic techniques, the potential of metabolomic methods in the typing of microorganisms has yet to be validated. Notably, the analysis of bacterial metabolites and/or metabolic pathways could provide information on the phenotype, allowing to deepen the knowledge of the biological functions of certain bacterial species. Up to now, the metabolome of lactobacilli has been investigated only by indirect methods, through genome-wide approaches based on the complete analysis of genes responsible for several metabolic pathways [4, 42, 53, 54]. The interesting novelty of the present taxonomic study lies on the direct metabolomic analysis by means of ^1^H-NMR of both extracellular and intracellular bacterial compartments.

It is worth emphasizing some important strengths of metabolomic analysis by ^1^H-NMR: (i) it is an intrinsically quantitative technique, which can avoid relying on internal standards [55], (ii) the experimental protocol is therefore extremely simple and quick, allowing processing tens of samples per batch, (iii) the method for sample preparation is the same used for the ribosomal sequences analysis, allowing excellent integration of the two techniques, (iv) the cost per analysis, at present slightly lower than gene sequencing, can be foreseen to drop dramatically in the short term [56].

From our results ^1^H-NMR analysis did not highlight specific metabolic profiles that could be univocally associated to the different *Lactobacillus* species or species groupings. This finding may be due to several aspects. ^1^H-NMR spectroscopy can detect only the most abundant metabolites, present at concentrations greater than 1 to 5 μM [57, 58]. Probably, the low sensitivity of this method led to identify only 47 molecules, considering globally the extracellular and intracellular *Lactobacillus* metabolome. In addition, differently from the high variability found in some regions of 16s rRNA gene and in the composition of ribosomal protein pattern, the metabolic traits can be more conserved among different species of the same bacterial genus. Indeed, a high variability in term of metabolic activity was observed among different strains of the same *Lactobacillus* species (i.e. lysine production by *L*. *delbrueckii* and the pyruvate production by *L*. *acidophilus*). This finding is in agreement with previous reports showing that closely related species can present marked differences in their metabolic traits: several metabolic pathways and molecules are associated with particular *Lactobacillus* species, while others are strain-specific rather than species-specific [4, 54]. Due to the metabolite concentration overlapping between different *Lactobacillus* species and the high intra-species variability, we cannot propose the metabolomic approach as an independent method for lactobacilli species identification. Nevertheless, it could represent a promising tool to study correlations with biological functions, allowing for example to predict the anti-microbial activity of *Lactobacillus* strains and to better understand the related mechanisms.

In fact, our results underline the high metabolic activity of *L*. *crispatus* strains in term of organic acids production and glucose consumption, compared to other *Lactobacillus* species. This aspect could probably have a connection with the biological activity shown by this species in vivo. Indeed, it is well known that *L*. *crispatus* strains possess a marked anti-microbial activity against several urogenital and sexually transmitted pathogens [28, 42, 59] and, recently, it has been shown that glucose depletion induced by *L*. *crispatus* is directly associated with the reduction of *Chlamydia trachomatis* infectivity [29].

We found that *L*. *casei*-*L*. *paracasei*-*L*. *rhamnosus* species were characterized by the highest production of lactate. In agreement with this finding, it has been recently described that *L*. *casei* is one of the dominant microbial species on different type of fruit residues and that it could play an important role during silage fermentation as a strong producer of lactic acid [60]. Moreover, the strong production of acetoin in *L*. *casei*-*L*. *paracasei*-*L*. *rhamnosus* species and the high increase of pyruvate in *L*. *acidophilus* species extracellular metabolome are in line with the results shown by Helland *et* al., regarding the growth and metabolism of selected strains of probiotic bacteria in maize porridge with added malted barley [61].

We are fully aware that a metabolomic approach based on the identification of molecules after bacterial cultivation, could be affected by the culture conditions (type of medium, incubation time, aerobic/anaerobic atmosphere). For that reason, a strict standardization of the culture parameters, as well as of the metabolite measurement, is mandatory.

## Conclusions

In conclusion, our study suggests novel approaches for the taxonomic and metabolic characterization of members of *Lactobacillus*. On one hand, as underlined by the excellent agreement with the reference genotypic method, MALDI-TOF MS is an outstanding technique for taxonomic purposes, thanks to its rapidity and simplicity. On the other hand, the metabolomic approach based on ^1^H-NMR analysis cannot be proposed as a reliable and powerful tool for the lactobacilli species identification. However, the ^1^H-NMR analysis led to identify a panel of molecules whose variations were strictly associated with the taxonomy. For that reason, it could be useful in correlating lactobacilli with biological properties, such as their anti-microbial activity or fermentation capacity for food production.

Further studies, comprising a larger number of strains and a broader panel of species, are needed to better elucidate the correlation between lactobacilli metabolome and taxonomy and to better assess how an integrated ‘multi-omics’ approach could help in a more accurate and predictive characterization of the *Lactobacillus* genus.

## Supporting information

S1 File **Table A. Metabolites identified by** ^**1**^**H-NMR in cell free supernatants of lactobacilli.** Concentrations were calculated as differences from MRS medium. Values are expressed as mmol/l. **Table B. Metabolites identified by** ^**1**^**H-NMR in bacterial lysates of lactobacilli strains.** Concentrations are expressed as mmol/l.
(DOCX)
