# Supplementary material for: Novel approaches for the taxonomic and metabolic characterization of lactobacilli: Integration of 16S rRNA gene sequencing with MALDI-TOF MS and 1H-NMR
Source: PLoS One. 2017 Feb 16;12(2):e0172483. doi: 10.1371/journal.pone.0172483 (PMC5312945; doi:10.1371/journal.pone.0172483)
Supplement: S1 File — Table A. Metabolites identified by 1H-NMR in cell free supernatants of lactobacilli. Concentrations were calculated as differences from MRS medium. Values are expressed as mmol/l. Table B. Metabolites identified by 1H-NMR in bacterial lysates of lactobacilli strains. Concentrations are expressed as mmol/l. (DOCX) [file pone.0172483.s001.docx]

**Table A.**

| **Strain** | **Molecule** | | | | | | | |
| --- | --- | --- | --- | --- | --- | --- | --- | --- |
|  | **Leucine** | **Isoleucine** | **Valine** | **Ethanol** | **Lactate** | **Alanine** | **Valerate** | **Acetate** |
| **MB233** | 1.185 | 0.278 | 0.352 | 0.239 | 2.659 | 0.394 | -0.099 | -26.689 |
| **MB422** | 3.266 | 0.504 | 0.842 | 0.408 | 1.803 | 1.104 | -0.058 | -36.663 |
| **MB423** | 4.359 | 0.671 | 1.165 | 0.272 | 2.008 | 1.897 | -0.038 | -56.623 |
| **DSM20079** | 1.154 | 0.274 | 0.457 | 0.307 | 18.346 | 1.083 | -0.076 | -23.196 |
| **LA14** | 4.185 | 0.732 | 1.284 | 0.470 | 0.383 | 1.184 | -0.036 | -52.119 |
| **CD2** | 0.749 | 0.356 | 0.359 | 12.515 | 6.286 | 0.463 | -0.072 | -26.495 |
| **DSM20011** | 1.536 | 0.459 | 0.686 | -0.023 | 27.538 | 0.563 | -0.100 | -28.565 |
| **BC1** | 1.665 | 0.366 | 0.508 | 2.077 | 18.467 | 0.542 | -0.092 | -23.576 |
| **BC3** | 2.548 | 0.661 | 0.878 | 0.005 | 15.516 | 1.581 | -0.068 | -27.592 |
| **BC4** | 1.972 | 0.385 | 0.552 | 0.013 | 17.042 | 0.853 | -0.075 | -22.403 |
| **BC5** | 1.578 | 0.319 | 0.421 | 0.015 | 14.046 | 0.625 | -0.070 | -29.818 |
| **BC6** | 1.828 | 0.541 | 0.640 | 0.520 | 12.394 | 1.223 | -0.063 | -36.260 |
| **BC7** | 2.000 | 0.358 | 0.526 | 0.023 | 11.897 | 0.728 | -0.070 | -23.666 |
| **BC8** | 3.132 | 0.548 | 0.944 | 0.221 | 0.907 | 1.326 | -0.048 | -54.809 |
| **DSM20081** | 2.998 | 0.562 | 0.898 | 0.992 | 0.719 | 1.056 | -0.048 | -51.325 |
| **DSM20074** | 2.028 | 0.601 | 0.967 | 0.069 | 4.738 | 0.450 | -0.045 | -45.870 |
| **DSM20076** | 0.966 | 0.608 | 0.829 | 0.344 | 30.726 | 0.423 | -0.070 | -26.530 |
| **FV13** | 1.112 | 0.334 | 0.426 | 1.125 | 7.536 | 0.438 | -0.071 | -26.652 |
| **BC9** | 3.506 | 0.567 | 1.011 | 0.004 | 1.504 | 1.280 | -0.037 | -51.844 |
| **BC10** | 1.531 | 0.492 | 0.638 | 4.894 | 6.948 | 0.931 | -0.069 | -28.890 |
| **BC11** | 3.762 | 0.689 | 1.197 | -0.038 | 2.850 | 1.545 | -0.050 | -46.245 |
| **BC12** | 0.922 | 0.354 | 0.440 | 0.157 | 16.436 | 0.662 | -0.073 | -28.902 |
| **BC13** | 2.970 | 0.721 | 1.127 | 0.442 | 3.426 | 1.631 | -0.041 | -51.493 |
| **BC14** | 1.378 | 0.306 | 0.397 | 0.191 | 29.168 | 0.568 | -0.069 | -25.749 |
| **DSM20243** | 3.822 | 0.622 | 1.115 | 0.518 | 1.762 | 1.378 | -0.040 | -49.971 |
| **LB31** | 2.779 | 0.922 | 1.461 | 0.734 | 8.695 | 2.664 | -0.053 | -46.309 |
| **LC10** | 0.844 | 0.311 | 0.476 | 0.256 | 41.156 | 0.997 | 0.004 | -32.398 |
| **DSM20314** | 1.111 | 0.332 | 0.406 | 0.335 | 18.228 | 0.441 | -0.077 | -24.402 |
| **BC18** | 0.543 | 0.290 | 0.363 | 2.931 | 19.441 | 0.470 | -0.079 | -22.895 |
| **BC19** | 0.482 | 0.269 | 0.346 | 1.240 | 38.830 | 0.426 | -0.103 | -20.565 |
| **BC20** | 2.461 | 0.447 | 0.691 | 0.485 | 3.327 | 0.866 | -0.061 | -43.108 |
| **DSM20174** | 2.932 | 0.586 | 0.938 | -0.002 | 7.671 | 1.418 | -0.048 | -42.062 |
| **FV9** | 0.885 | 0.320 | 0.379 | 0.033 | 23.170 | 0.599 | -0.089 | -21.127 |
| **LPT** | 1.338 | 0.363 | 0.492 | 0.124 | 18.467 | 0.520 | -0.095 | -27.655 |
| **MB313** | 1.974 | 0.558 | 0.819 | 39.305 | 10.328 | 1.203 | -0.072 | -30.900 |
| **DSM20016** | 0.808 | 0.459 | 0.651 | 34.216 | 21.221 | 1.118 | -0.080 | -25.500 |
| **B876** | 2.424 | 0.438 | 0.800 | 0.579 | 7.753 | 1.447 | -0.067 | -44.672 |
| **DSM20021** | 1.508 | 0.369 | 0.518 | 0.336 | 18.664 | 0.795 | -0.075 | -25.504 |
| **BC16** | 1.441 | 0.605 | 0.777 | 29.307 | 16.148 | 1.037 | -0.095 | -25.639 |
| **BC17** | 5.383 | 1.318 | 1.374 | 68.386 | 21.821 | 1.561 | -0.001 | -35.510 |

| **Strain** | **Molecule** | | | | | | | |
| --- | --- | --- | --- | --- | --- | --- | --- | --- |
|  | **Methionine** | **Acetoin** | **Acetone** | **Pyruvate** | **Sarcosine** | **Aspartate** | **Choline** | **sn-glycero-3-phospho**  **choline** |
| **MB233** | 0.436 | 0.015 | 0.028 | 0.198 | 0.055 | 0.023 | -0.049 | -0.204 |
| **MB422** | 0.745 | 0.020 | 0.030 | 0.393 | 0.055 | -0.019 | 0.000 | -0.173 |
| **MB423** | 0.967 | 0.017 | 0.040 | 1.355 | 0.041 | -0.033 | 0.027 | -0.150 |
| **DSM20079** | 0.462 | 0.019 | 0.040 | 1.409 | 0.069 | 0.050 | -0.041 | -0.188 |
| **LA14** | 0.983 | 0.001 | 0.043 | 0.883 | 0.048 | -0.033 | 0.017 | -0.153 |
| **CD2** | 0.258 | 0.002 | -0.016 | 0.098 | 0.106 | 0.060 | -0.062 | -0.211 |
| **DSM20011** | 0.578 | 0.086 | -0.012 | 0.180 | -0.014 | 0.081 | -0.053 | -0.194 |
| **BC1** | 0.559 | 0.000 | -0.020 | 0.155 | 0.018 | 0.026 | -0.044 | -0.188 |
| **BC3** | 0.759 | 0.114 | -0.009 | 0.785 | 0.010 | 0.039 | -0.025 | -0.156 |
| **BC4** | 0.635 | -0.001 | -0.012 | 0.201 | 0.004 | 0.033 | -0.027 | -0.193 |
| **BC5** | 0.513 | 0.001 | -0.002 | 0.420 | 0.019 | 0.019 | -0.032 | -0.191 |
| **BC6** | 0.458 | 0.037 | 0.004 | 0.378 | 0.082 | 0.014 | -0.039 | -0.173 |
| **BC7** | 0.609 | -0.001 | 0.007 | 0.236 | 0.016 | 0.017 | -0.020 | -0.177 |
| **BC8** | 0.624 | -0.018 | 0.035 | 0.114 | 0.070 | -0.034 | -0.002 | -0.176 |
| **DSM20081** | 0.507 | -0.013 | 0.021 | 0.153 | 0.141 | -0.029 | 0.011 | -0.187 |
| **DSM20074** | 0.562 | -0.003 | 0.028 | 0.187 | 0.068 | -0.017 | 0.011 | -0.287 |
| **DSM20076** | 0.510 | 0.050 | -0.015 | 0.254 | 0.037 | 0.074 | -0.085 | -0.230 |
| **FV13** | 0.282 | 0.009 | 0.014 | 0.169 | 0.110 | 0.032 | -0.043 | -0.233 |
| **BC9** | 0.749 | 0.347 | -0.008 | 0.133 | 0.030 | -0.030 | 0.013 | -0.156 |
| **BC10** | 0.531 | -0.004 | -0.028 | 0.079 | 0.028 | 0.031 | -0.064 | -0.190 |
| **BC11** | 0.850 | -0.012 | -0.009 | 0.108 | 0.034 | -0.022 | 0.014 | -0.121 |
| **BC12** | 0.349 | 0.012 | 0.011 | 0.253 | 0.084 | 0.046 | -0.053 | -0.190 |
| **BC13** | 0.558 | -0.018 | 0.018 | 0.168 | 0.129 | -0.028 | 0.001 | -0.169 |
| **BC14** | 0.504 | 0.003 | 0.010 | 0.564 | 0.046 | 0.028 | -0.035 | -0.196 |
| **DSM20243** | 0.758 | -0.003 | 0.016 | 0.088 | 0.092 | -0.029 | 0.014 | -0.158 |
| **LB31** | 0.788 | -0.012 | -0.030 | 0.198 | 0.126 | 0.011 | -0.031 | -0.169 |
| **LC10** | 0.481 | 0.373 | -0.035 | 0.476 | -0.016 | 0.106 | -0.072 | -0.213 |
| **DSM20314** | 0.413 | 0.039 | -0.016 | 0.169 | 0.039 | 0.054 | -0.079 | -0.207 |
| **BC18** | 0.255 | 0.001 | -0.008 | 0.108 | 0.096 | 0.033 | -0.107 | -0.205 |
| **BC19** | 0.393 | 0.005 | -0.030 | 0.105 | -0.017 | 0.065 | -0.110 | -0.197 |
| **BC20** | 0.578 | 0.008 | 0.007 | 0.105 | 0.006 | -0.017 | -0.081 | -0.189 |
| **DSM20174** | 0.667 | 0.037 | 0.000 | 0.236 | -0.007 | 0.004 | -0.063 | -0.159 |
| **FV9** | 0.477 | 0.028 | -0.012 | 0.114 | -0.007 | 0.055 | -0.085 | -0.201 |
| **LPT** | 0.522 | 0.024 | -0.002 | 0.126 | -0.011 | 0.035 | -0.092 | -0.193 |
| **MB313** | 0.453 | -0.015 | -0.040 | 0.140 | 0.060 | 0.001 | -0.022 | -0.199 |
| **DSM20016** | 0.271 | -0.004 | -0.009 | 0.161 | 0.096 | 0.041 | -0.044 | -0.214 |
| **B876** | 0.610 | 0.121 | 0.011 | 0.228 | 0.018 | -0.012 | 0.005 | -0.168 |
| **DSM20021** | 0.500 | 0.287 | -0.006 | 0.211 | 0.044 | 0.042 | -0.027 | -0.207 |
| **BC16** | 0.395 | -0.006 | -0.031 | 0.120 | 0.058 | 0.060 | -0.108 | -0.214 |
| **BC17** | 0.317 | -0.001 | 0.023 | 0.312 | 0.064 | -0.002 | -0.028 | -0.219 |

| **Strain** | **Molecule** | | | | | | | |
| --- | --- | --- | --- | --- | --- | --- | --- | --- |
|  | **Pyro**  **glutamate** | **1.3-dihydroxy**  **acetone** | **Lactose** | **Ribose** | **Glucose** | **Uracil** | **Uridine** | **Cytosine** |
| **MB233** | -0.879 | 0.135 | -0.480 | 0.024 | -7.398 | -0.049 | -0.015 | 0.013 |
| **MB422** | -0.528 | 0.297 | 0.082 | 0.016 | -5.124 | 0.066 | -0.004 | 0.025 |
| **MB423** | -1.497 | 0.491 | -0.603 | 0.038 | -5.749 | -0.022 | -0.040 | 0.005 |
| **DSM20079** | -0.219 | 0.391 | -1.078 | 0.070 | -10.983 | 0.017 | -0.005 | 0.058 |
| **LA14** | -0.596 | -0.001 | -1.148 | 0.044 | -9.166 | 0.039 | -0.006 | -0.006 |
| **CD2** | -0.985 | 0.010 | -1.057 | 0.053 | -12.874 | 0.119 | -0.068 | 0.022 |
| **DSM20011** | -0.255 | 0.017 | -0.551 | 0.030 | -11.485 | -0.048 | 0.011 | 0.008 |
| **BC1** | -0.072 | 0.295 | -0.601 | 0.055 | -11.424 | -0.057 | -0.008 | 0.024 |
| **BC3** | -0.552 | 0.345 | -0.230 | 0.145 | -8.569 | -0.034 | -0.021 | 0.118 |
| **BC4** | -0.583 | 0.209 | -0.398 | 0.106 | -8.803 | -0.071 | -0.012 | 0.048 |
| **BC5** | -0.700 | 0.252 | -0.404 | 0.073 | -9.674 | -0.064 | -0.009 | 0.031 |
| **BC6** | -1.019 | 0.265 | -0.491 | 0.206 | -9.734 | -0.043 | -0.026 | 0.070 |
| **BC7** | -0.597 | 0.193 | -0.396 | 0.028 | -7.494 | -0.045 | -0.006 | 0.013 |
| **BC8** | -0.980 | 0.467 | 0.113 | 0.067 | -8.752 | 0.003 | -0.028 | 0.004 |
| **DSM20081** | -1.011 | 0.199 | -2.106 | 0.013 | -4.312 | 0.016 | -0.025 | 0.019 |
| **DSM20074** | -0.192 | 0.588 | 0.034 | 0.007 | -12.128 | -0.015 | -0.067 | 0.019 |
| **DSM20076** | -0.017 | 0.091 | -1.793 | 0.024 | -13.473 | -0.007 | -0.063 | 0.001 |
| **FV13** | -0.208 | 0.266 | -1.067 | 0.032 | -10.187 | -0.022 | -0.039 | 0.005 |
| **BC9** | -1.220 | 0.490 | 0.130 | 0.002 | -8.450 | -0.032 | -0.018 | -0.002 |
| **BC10** | -0.108 | 0.198 | -0.738 | 0.079 | -8.806 | -0.021 | -0.038 | 0.004 |
| **BC11** | -0.874 | 0.380 | 0.201 | 0.041 | -2.564 | 0.031 | -0.012 | 0.031 |
| **BC12** | -0.153 | 0.288 | -0.643 | 0.075 | -14.058 | -0.008 | -0.040 | 0.043 |
| **BC13** | 0.113 | 1.007 | -0.154 | 0.052 | -18.606 | 0.013 | -0.049 | -0.036 |
| **BC14** | -0.660 | 0.445 | -0.420 | 0.099 | -15.367 | -0.082 | -0.005 | 0.050 |
| **DSM20243** | -0.471 | 0.425 | 0.111 | 0.005 | -8.196 | -0.044 | 0.007 | 0.009 |
| **LB31** | -1.154 | 0.756 | -1.696 | 0.196 | -10.107 | 0.086 | -0.061 | 0.113 |
| **LC10** | -0.212 | 0.052 | -0.415 | 0.017 | -12.948 | -0.138 | -0.009 | -0.014 |
| **DSM20314** | -0.263 | 0.017 | -0.366 | 0.025 | -10.399 | -0.050 | -0.023 | 0.004 |
| **BC18** | -0.154 | 0.330 | -0.993 | 0.024 | -14.668 | -0.067 | -0.021 | -0.002 |
| **BC19** | -0.196 | 0.253 | -0.834 | 0.025 | -13.913 | -0.076 | -0.033 | -0.007 |
| **BC20** | -1.258 | 0.044 | 0.026 | 0.067 | -8.593 | -0.038 | -0.024 | -0.012 |
| **DSM20174** | -0.329 | 0.043 | 0.182 | 0.014 | -10.738 | -0.070 | -0.020 | -0.019 |
| **FV9** | -0.203 | 0.201 | -0.667 | 0.019 | -9.295 | -0.019 | -0.011 | 0.016 |
| **LPT** | -0.158 | 0.014 | -0.535 | 0.021 | -11.162 | -0.060 | -0.022 | 0.024 |
| **MB313** | -0.111 | -0.048 | -1.449 | 0.064 | -17.265 | 0.012 | -0.047 | -0.012 |
| **DSM20016** | -0.137 | 0.005 | -2.142 | 0.101 | -21.482 | -0.010 | -0.062 | -0.001 |
| **B876** | -1.014 | 0.013 | 0.420 | 0.003 | -6.881 | -0.079 | 0.020 | -0.023 |
| **DSM20021** | -0.394 | 0.023 | -0.122 | 0.009 | -10.933 | -0.049 | 0.009 | -0.001 |
| **BC16** | -0.245 | 0.119 | -1.681 | 0.102 | -18.401 | -0.094 | -0.015 | -0.022 |
| **BC17** | -0.130 | 0.184 | -3.529 | 0.048 | -35.389 | -0.125 | 0.004 | -0.026 |

| **Strain** | **Molecule** | | | | | |
| --- | --- | --- | --- | --- | --- | --- |
|  | **Orotate** | **Tyrosine** | **Tryptophan** | **Phenylalanine** | **Formate** | **Butyrate** |
| **MB233** | 0.003 | -0.192 | -0.454 | 0.464 | 0.051 | -1.109 |
| **MB422** | 0.003 | 0.096 | -0.547 | 1.424 | -0.232 | -1.108 |
| **MB423** | 0.005 | 0.392 | -0.308 | 1.948 | -0.247 | -0.995 |
| **DSM20079** | 0.008 | -0.182 | -0.434 | 0.117 | 0.105 | -1.093 |
| **LA14** | 0.009 | 0.393 | -0.428 | 2.022 | -0.240 | -1.041 |
| **CD2** | 0.013 | -0.150 | -0.642 | 0.284 | 0.305 | -1.087 |
| **DSM20011** | 0.011 | 0.127 | -0.622 | 0.614 | -0.023 | -1.060 |
| **BC1** | 0.005 | -0.019 | -0.305 | 0.557 | -0.081 | -1.082 |
| **BC3** | 0.003 | 0.249 | -0.432 | 0.700 | -0.060 | -1.067 |
| **BC4** | 0.008 | 0.105 | -0.264 | 0.666 | -0.115 | -1.115 |
| **BC5** | 0.009 | -0.096 | -0.351 | 0.449 | -0.096 | -1.083 |
| **BC6** | 0.001 | 0.161 | -0.508 | 0.481 | -0.087 | -1.035 |
| **BC7** | 0.006 | 0.013 | -0.624 | 0.752 | -0.114 | -1.117 |
| **BC8** | 0.001 | 0.098 | -0.609 | 1.249 | -0.232 | -1.054 |
| **DSM20081** | -0.004 | 0.161 | -0.630 | 1.609 | -0.240 | -1.055 |
| **DSM20074** | 0.014 | 0.188 | -0.497 | 1.394 | -0.243 | -1.044 |
| **DSM20076** | 0.006 | 0.072 | -0.441 | 0.541 | 0.148 | -1.067 |
| **FV13** | 0.005 | -0.125 | -0.417 | 0.461 | 0.062 | -1.104 |
| **BC9** | 0.004 | 0.157 | -0.240 | 1.432 | -0.226 | -1.039 |
| **BC10** | -0.001 | 0.131 | -0.352 | 0.684 | -0.119 | -1.078 |
| **BC11** | 0.005 | 0.471 | -0.539 | 2.127 | -0.237 | -1.072 |
| **BC12** | 0.001 | -0.253 | -0.697 | 0.135 | 0.083 | -1.095 |
| **BC13** | -0.002 | 0.180 | -0.332 | 0.845 | -0.240 | -0.974 |
| **BC14** | 0.007 | -0.190 | -0.381 | 0.103 | 0.021 | -1.038 |
| **DSM20243** | 0.006 | 0.272 | -0.540 | 1.916 | -0.255 | -1.055 |
| **LB31** | -0.004 | 1.049 | -0.607 | 1.313 | -0.244 | -0.990 |
| **LC10** | 0.022 | -0.065 | -0.316 | 0.164 | 0.001 | -0.881 |
| **DSM20314** | 0.008 | -0.124 | -0.371 | 0.375 | 0.000 | -1.094 |
| **BC18** | 0.015 | -0.288 | -0.480 | -0.193 | 0.038 | -0.995 |
| **BC19** | 0.018 | -0.271 | -0.319 | -0.248 | 0.031 | -1.020 |
| **BC20** | 0.013 | 0.045 | -0.488 | 1.106 | -0.237 | -1.082 |
| **DSM20174** | 0.013 | 0.292 | -0.177 | 1.231 | -0.217 | -0.984 |
| **FV9** | 0.010 | -0.172 | -0.370 | 0.141 | 0.001 | -1.064 |
| **LPT** | 0.008 | -0.044 | -0.299 | 0.330 | -0.104 | -1.076 |
| **MB313** | 0.013 | 0.036 | -0.542 | 0.835 | -0.177 | -0.998 |
| **DSM20016** | 0.011 | -0.084 | -0.627 | 0.134 | -0.008 | -0.943 |
| **B876** | 0.005 | 0.260 | -0.513 | 1.324 | -0.215 | -0.997 |
| **DSM20021** | 0.012 | 0.024 | -0.618 | 0.683 | -0.055 | -1.053 |
| **BC16** | 0.000 | 0.098 | -0.650 | 0.531 | 0.022 | -1.006 |
| **BC17** | -0.009 | -0.044 | -0.733 | 0.383 | -0.167 | -0.849 |

**Table B.**

| **Strain** | **Molecule** | | | | | | | | |
| --- | --- | --- | --- | --- | --- | --- | --- | --- | --- |
|  | **Lactate** | **Acetate** | **Propionate** | **FADH** | **Ethanol** | **Pyruvate** | **Uracil** | **Uridine-diP**  **galactose** | **Lysine** |
| **MB233** | 1.691 | 0.641 | 0.401 | 0.001 | 0.285 | 0.017 | 0.004 | 0.024 | 0.005 |
| **MB422** | 2.298 | 1.263 | 0.126 | 0.007 | 0.529 | 0.070 | 0.012 | 0.097 | 0.068 |
| **MB423** | 4.968 | 1.399 | 0.107 | 0.007 | 0.212 | 0.131 | 0.005 | 0.021 | 0.038 |
| **DSM20079** | 3.445 | 1.389 | 0.080 | 0.006 | 0.375 | 0.119 | 0.003 | 0.088 | 0.049 |
| **LA14** | 1.985 | 1.277 | 0.089 | 0.013 | 0.379 | 0.115 | 0.012 | 0.088 | 0.191 |
| **CD2** | 2.956 | 3.167 | 0.048 | 0.007 | 1.844 | 0.040 | 0.022 | 0.022 | 0.047 |
| **DSM20011** | 14.196 | 3.341 | 0.123 | 0.013 | 0.770 | 0.008 | 0.044 | 0.002 | 0.011 |
| **BC1** | 5.331 | 2.635 | 0.182 | 0.006 | 1.142 | 0.006 | 0.022 | 0.026 | 0.042 |
| **BC3** | 5.418 | 2.112 | 0.178 | 0.005 | 1.128 | 0.006 | 0.013 | 0.029 | 0.032 |
| **BC4** | 4.620 | 2.903 | 0.200 | 0.003 | 1.116 | 0.006 | 0.014 | 0.016 | 0.038 |
| **BC5** | 4.105 | 1.717 | 0.209 | 0.006 | 1.114 | 0.016 | 0.011 | 0.026 | 0.026 |
| **BC6** | 5.441 | 2.096 | 0.539 | 0.006 | 0.130 | 0.200 | 0.029 | 0.015 | 0.031 |
| **BC7** | 2.776 | 1.779 | 0.231 | 0.011 | 1.230 | 0.023 | 0.019 | 0.037 | 0.031 |
| **BC8** | 5.141 | 1.422 | 0.165 | 0.002 | 0.353 | 0.069 | 0.006 | 0.009 | 0.055 |
| **DSM20081** | 8.684 | 2.131 | 0.061 | 0.005 | 0.191 | 0.515 | 0.003 | 0.051 | 0.151 |
| **DSM20074** | 3.734 | 2.806 | 0.105 | 0.000 | 0.373 | 0.131 | 0.002 | 0.024 | 0.092 |
| **DSM20076** | 1.875 | 3.313 | 0.082 | 0.003 | 0.225 | 0.155 | 0.012 | 0.029 | 0.202 |
| **FV13** | 5.442 | 2.466 | 0.036 | 0.004 | 0.839 | 0.007 | 0.008 | 0.023 | 0.071 |
| **BC9** | 5.981 | 1.775 | 0.177 | 0.001 | 1.106 | 0.024 | 0.005 | 0.017 | 0.022 |
| **BC10** | 3.426 | 2.072 | 0.156 | 0.002 | 0.981 | 0.010 | 0.005 | 0.018 | 0.023 |
| **BC11** | 3.531 | 1.983 | 0.144 | 0.001 | 1.018 | 0.013 | 0.003 | 0.018 | 0.051 |
| **BC12** | 4.621 | 2.419 | 0.122 | 0.004 | 0.441 | 0.022 | 0.013 | 0.020 | 0.108 |
| **BC13** | 7.236 | 2.326 | 0.098 | 0.006 | 0.315 | 0.076 | 0.017 | 0.023 | 0.026 |
| **BC14** | 7.586 | 3.084 | 0.242 | 0.009 | 0.125 | 0.391 | 0.033 | 0.028 | 0.027 |
| **DSM20243** | 2.432 | 1.798 | 0.235 | 0.001 | 0.579 | 0.012 | 0.017 | 0.009 | 0.066 |
| **LB31** | 3.355 | 1.304 | 0.155 | 0.009 | 0.759 | 0.026 | 0.009 | 0.003 | 0.010 |
| **LC10** | 14.447 | 2.976 | 0.094 | 0.002 | 0.419 | 0.023 | 0.003 | 0.032 | 0.044 |
| **DSM20314** | 1.827 | 0.860 | 0.193 | -0.001 | 0.698 | 0.000 | 0.011 | 0.010 | 0.003 |
| **BC18** | 13.842 | 3.490 | 0.058 | 0.006 | 0.993 | 0.124 | 0.003 | 0.041 | 0.059 |
| **BC19** | 15.317 | 3.408 | 0.129 | 0.005 | 0.968 | 0.121 | 0.015 | 0.034 | 0.033 |
| **BC20** | 3.736 | 1.221 | 0.159 | -0.001 | 0.955 | 0.007 | 0.009 | 0.017 | 0.035 |
| **DSM20174** | 11.296 | 2.158 | 0.158 | 0.000 | 0.830 | 0.069 | 0.010 | 0.015 | 0.027 |
| **FV9** | 13.644 | 1.950 | 0.153 | 0.007 | 0.978 | 0.139 | 0.010 | 0.023 | 0.019 |
| **LPT** | 4.942 | 1.213 | 0.005 | 0.001 | 0.451 | 0.058 | 0.009 | 0.042 | 0.004 |
| **MB313** | 7.081 | 2.677 | 0.291 | 0.011 | 0.204 | 0.027 | 0.049 | 0.008 | 0.016 |
| **DSM20016** | 3.519 | 1.798 | 0.123 | 0.004 | 1.041 | 0.031 | 0.041 | 0.009 | 0.007 |
| **B876** | 7.833 | 1.386 | 0.377 | 0.001 | 0.219 | 0.010 | 0.007 | 0.040 | 0.020 |
| **DSM20021** | 5.964 | 2.649 | 0.139 | 0.001 | 0.588 | 0.019 | 0.102 | 0.034 | 0.040 |
| **BC16** | 2.650 | 2.149 | 0.222 | 0.005 | 1.469 | 0.005 | 0.006 | 0.009 | 0.030 |
| **BC17** | 9.736 | 8.015 | 0.085 | 0.002 | 1.593 | 0.031 | 0.013 | 0.007 | 0.057 |

| **Strain** | **Molecule** | | | | | | | |
| --- | --- | --- | --- | --- | --- | --- | --- | --- |
|  | **Succinate** | **Aspartate** | **Isoleucine** | **Valine** | **Acetone** | **AMP** | **Isovalerate** | **NADplus** |
| **MB233** | 0.048 | 0.046 | 0.181 | 0.067 | 0.050 | 0.011 | 0.020 | 0.015 |
| **MB422** | 0.259 | 0.176 | 0.070 | 0.035 | 0.030 | 0.040 | 0.013 | 0.019 |
| **MB423** | 0.233 | 0.052 | 0.132 | 0.079 | 0.006 | 0.019 | 0.023 | 0.049 |
| **DSM20079** | 0.163 | 0.108 | 0.195 | 0.075 | 0.024 | 0.045 | 0.034 | 0.049 |
| **LA14** | 0.071 | 0.085 | 0.104 | 0.052 | 0.036 | 0.026 | 0.027 | 0.013 |
| **CD2** | 0.059 | 0.097 | 0.073 | 0.051 | 0.109 | -0.002 | 0.031 | 0.033 |
| **DSM20011** | 0.099 | 0.048 | 0.248 | 0.117 | 0.069 | 0.004 | 0.019 | 0.006 |
| **BC1** | 0.212 | 0.057 | 0.159 | 0.090 | 0.011 | 0.056 | 0.025 | 0.053 |
| **BC3** | 0.361 | 0.020 | 0.085 | 0.061 | 0.008 | 0.017 | 0.009 | 0.053 |
| **BC4** | 0.269 | 0.036 | 0.172 | 0.090 | 0.008 | 0.029 | 0.005 | 0.053 |
| **BC5** | 0.126 | 0.039 | 0.150 | 0.080 | 0.010 | 0.053 | 0.010 | 0.067 |
| **BC6** | 0.234 | 0.012 | 0.065 | 0.029 | 0.065 | 0.040 | 0.024 | 0.042 |
| **BC7** | 0.110 | 0.026 | 0.122 | 0.072 | 0.009 | 0.021 | 0.004 | 0.037 |
| **BC8** | 0.128 | 0.022 | 0.137 | 0.073 | 0.002 | 0.023 | 0.013 | 0.048 |
| **DSM20081** | 0.136 | 0.078 | 0.103 | 0.066 | 0.084 | 0.013 | 0.073 | 0.003 |
| **DSM20074** | 0.125 | 0.102 | 0.095 | 0.057 | 0.013 | 0.016 | 0.020 | 0.013 |
| **DSM20076** | 0.075 | 0.114 | 0.167 | 0.075 | 0.012 | 0.011 | 0.033 | 0.006 |
| **FV13** | 0.117 | 0.023 | 0.071 | 0.050 | 0.110 | 0.005 | 0.056 | 0.047 |
| **BC9** | 0.056 | 0.012 | 0.096 | 0.068 | 0.011 | 0.008 | 0.005 | 0.016 |
| **BC10** | 0.127 | 0.079 | 0.137 | 0.078 | 0.043 | 0.021 | 0.017 | 0.012 |
| **BC11** | 0.085 | 0.044 | 0.154 | 0.091 | 0.005 | 0.010 | 0.008 | 0.037 |
| **BC12** | 0.132 | 0.050 | 0.173 | 0.094 | 0.007 | 0.026 | 0.042 | 0.051 |
| **BC13** | 0.193 | 0.016 | 0.164 | 0.090 | 0.004 | 0.033 | 0.033 | 0.051 |
| **BC14** | 0.315 | 0.040 | 0.109 | 0.069 | 0.007 | 0.118 | 0.012 | 0.056 |
| **DSM20243** | 0.024 | 0.042 | 0.059 | 0.033 | 0.034 | 0.050 | 0.017 | 0.013 |
| **LB31** | 0.097 | 0.009 | 0.059 | 0.049 | 0.147 | 0.002 | 0.096 | 0.004 |
| **LC10** | 0.095 | 0.029 | 0.137 | 0.079 | 0.011 | -0.003 | 0.040 | 0.011 |
| **DSM20314** | 0.018 | 0.009 | 0.013 | 0.010 | 0.036 | 0.007 | 0.029 | 0.008 |
| **BC18** | 0.492 | 0.079 | 0.075 | 0.037 | 0.018 | 0.006 | 0.029 | 0.020 |
| **BC19** | 0.229 | 0.062 | 0.060 | 0.034 | 0.029 | 0.008 | 0.018 | 0.025 |
| **BC20** | 0.017 | 0.005 | 0.035 | 0.028 | 0.040 | 0.003 | 0.023 | 0.004 |
| **DSM20174** | 0.030 | 0.019 | 0.074 | 0.044 | 0.047 | 0.007 | 0.050 | 0.014 |
| **FV9** | 0.043 | 0.036 | 0.025 | 0.021 | 0.053 | 0.008 | 0.030 | 0.030 |
| **LPT** | 0.034 | 0.002 | 0.024 | 0.008 | 0.019 | 0.002 | 0.004 | 0.013 |
| **MB313** | 0.223 | 0.039 | 0.095 | 0.031 | 0.014 | 0.000 | 0.012 | 0.006 |
| **DSM20016** | 0.055 | 0.112 | 0.094 | 0.043 | 0.010 | 0.002 | 0.024 | 0.002 |
| **B876** | 0.081 | 0.022 | 0.029 | 0.016 | 0.041 | 0.011 | 0.021 | 0.041 |
| **DSM20021** | 0.095 | 0.028 | 0.115 | 0.061 | 0.055 | 0.012 | 0.048 | 0.028 |
| **BC16** | 0.063 | 0.100 | 0.115 | 0.048 | 0.031 | 0.013 | 0.047 | 0.004 |
| **BC17** | 0.395 | 0.056 | 0.148 | 0.067 | 0.048 | 0.017 | 0.033 | 0.002 |
